# Topics of the Month

**Published:** 1897-02

**Authors:** 


### TOPICS OF THE MONTH.

THE superintendent of the streets of Buffalo, Mr. T. F. Maloney, is entitled to great credit for the manner in which he has dealt with the snow problem on Main street during the first part of the present winter season. With the first fall of snow he promptly put a force of men and teams upon the street and cleared out the bulk of snow that had fallen, leaving simply a thin coat to facilitate traffic ; and this he followed up after each successive fall of snow, so that thus far our principal business thoroughfare has been kept in much better condition than usual in winter. This has the treble effect of facilitating traffic, furnishing employment to the needy and contributing to improve the health of the city. We have heretofore pointed out that one of the ways to prevent disease is to employ idle hands, and we are happy to note that our suggestions apparently have taken root in the minds of our municipal executive and administrative officers.

---

THE effect of good government in the city of New York is strikingly shown in the lowering of the death-rate for the last year, the credit for which must be awarded mainly to the efficiency of the health department and the department of street cleaning. It is an important fact to observe that these two departments go hand in hand in whatever results that are beneficial or harmful to the health interests of a city. In other words, it is important that these two branches of municipal government keep in close touch in order to maintain a high standard of healthfulness.

---

REFORMS in suppressing unnecessary noises are going forward in various cities. The district commissioners of Washington, D. C., announce that on and after February 1, 1897, newsboys must cease to shout their wares within the city limits on Sunday. This is one of the most commendable actions lately taken in any city. The nuisance is growing to be abominable. Between the early shoutings of local newspapers and the late afternoon crying of the New York dailies, the streets of Buffalo are given over to intolerable noise on Sunday. It is quite time for the Police Department to interfere and suppress it.

Indianapolis, a city that has increased over 10,000 inhabitants within the past year, and has now grown to be one of the large cities of the land, has recently passed an ordinance prohibiting the blowing of tin horns or other noise-making instruments. An attempt was made to except holiday week and legal holidays, but this was voted down, and a fine not to exceed $50 or thirty days' imprisonment, one or both, made the penalty. The examples of Washington and Indianapolis are worthy of emulation in Buffalo.

---

A STATUE of the late Dr. Samuel D. Gross, professor of surgery at Jefferson Medical College for twenty-six years prior to 1882, has recently been cast at Paris, and is expected to arrive in this country the present month. It will be erected on the grounds of the Smithsonian Institution at Washington.

The movement for the erection of this statue was begun by Dr. Claudius H. Mastin, of Mobile, who introduced a resolution in favor of the project at the meeting of the American Surgical Association, at Washington, September, 1891. Dr. Mastin was then president of the association and devoted a portion of his annual address to this subject.

Dr. Gross was one of the founders and the first president of the surgical association, hence this was a fitting place in which to take the initiative for so commendable a project. The statue is by Mr. A. Sterling Calder, a student of the art school of the academy of fine arts. Mr. Calder went abroad and modeled the statue in Paris, where it has been cast in bronze. Congress has appropriated $1,500 for the pedestal, and the statue is to be placed near the army medical museum. This monument will be the second of the kind erected to a physician in this country, the other being that of Dr. J. Marion Sims, at New York. Both of these men were graduates of Jefferson Medical College, and among its most distinguished alumni.

The statue will be unveiled by a granddaughter of Dr. Gross next May, during the triennial congress of American Physicians and Surgeons at Washington.

---

IN THE street cars of Philadelphia there is a sign that reads, "Spitting on the floor of this car is prohibited, by order of the board of health." In Boston a similar notice reads as follows: "The board of health adjudges that the deposit of sputum in street cars is a public nuisance, a source of filth, and a cause of sickness, and hereby orders that the spitting on the floors of street cars be, and hereby is, prohibited." Above this order is posted a copy of the statute that somewhat sententiously states that the fine for infringement of an order of the board of health is $100. These official acts by the boards of health of Philadelphia and Boston, respectively, are indicative of a progress that is making along the lines of cleanliness and preventive medicine. If we mistake not, the propriety of similar action was first proposed by the health department of Buffalo, and we are pleased to see that other cities have even taken more decided action than we have yet been able to enforce or maintain here.

---

THIS JOURNAL is willing to accept subscriptions to the proposed monument of Pasteur, as indicated in our issue of August, 1896. Brigadier-General George M. Sternberg, M. D., surgeon-general U. S. Army, is treasurer. Dr. A. Dagenais, of Buffalo, has subscribed $2.00, and we shall be glad to record and transmit the subscriptions of any others who may feel willing to contribute to this commendable object.

---

THE health department of the city of New York has lately made public a report of one of its committees with relation to the controlling of consumption. It is one of the most important reports that has been published on the subject and we call attention to some of its features. It states that there are now about 20,000 cases of pulmonary tuberculosis in that city, and that from thirty to fifty of its inhabitants daily become affected with the disease, one-half of whom die, and further that nearly all of those suffering death might be prevented by the application of scientific knowledge and sanitary regulations. It reviews the work done by the health department with the limited resources at its command, and declares that the time has now arrived when radical and comprehensive measures should be adopted to diminish the prevalence of the malady.

It further says that the knowledge now at command regarding the methods of extension of pulmonary tuberculosis, entirely justifies the belief that its ravages can certainly be limited by proper sanitary control and appropriate treatment, as can other infectious diseases. It asks for more power to enable the health department to cope with the ravages of the dreaded malady, and adds that the health authorities are convinced that no other factor is so potent to-day in perpetuating that ominous death-list from pulmonary tuberculosis, as the lack of proper facilities for the care of the poor stricken with the malady. The report ably deals with the question of the necessity of providing a special hospital for the treatment of patients afflicted with this disease, and concludes with the following recommendations:

1. That such action be taken by the health board as seems necessary and proper to at once secure the provision of hospital accommodations, under its charge, for the care of the poor suffering from pulmonary tuberculosis, who, as active sources of danger to the community, may properly come under its supervision.

2. That an amendment be made to the Sanitary Code, declaring that tuberculosis be officially considered a communicable disease, and formulating regulations under which its sanitary surveillance shall be exercised.

3. That all institutions in this city which admit and treat cases of pulmonary tuberculosis be subjected to regular and systematic inspection by officials of this board, and that specific regulations be established for the conduct of such institutions, in accord with the proposed amendment to the Sanitary Code.

4. That the scope of the measures designed for the education of the people in regard to the nature of pulmonary tuberculosis, and the methods to be taken for its prevention, be enlarged and a closer sanitary supervision be maintained over individuals suffering from this disease in the densely populated tenement districts and in the crowded workshops and public buildings of this city.

The report is signed by Herman M. Biggs, T. Mitchell Prudden and George B. Fowler.

General Emmons Clark, secretary of the health board, is quoted as having said that no immediate steps could be taken toward the erection of a hospital for consumptives, as the board of estimate had already made its appropriations for the year, and the passage of a legislative act will be required. The board will, however, he added, at its next meeting adopt an amendment to the sanitary code in line with the recommendations of the report.

---

THE antivivisectionists are carrying on a lively campaign in the halls of congress. The senate committee on the District of Columbia has issued a printed report to accompany senate bill 1552, which consists of a brochure of pp. xxiv.—159. It is a document that furnishes some interesting reading, pro and con, on the subject of vivisection. The bill referred to has the deceptive title, "For the further prevention of cruelty to animals in the District of Columbia." The friends of vivisection believe that it is so adroitly worded as to very much cripple original laboratory investigations, both in the colleges and in the government department situated in the District of Columbia, and that if it should pass it will be made a wedge to secure similar and even more restricted legislation in the states and other territories.

One of the most dignified, scholarly and forceful protests against the passage of the bill is made by the Association of American Physicians, and is signed by the members present at the last annual meeting. It is one of the most attractive and readable papers that we have seen on the subject, and it would benefit, if not instruct, everyone who would read it.

Among others who write letters favoring the passage of the bill we note that Dr. W. S. Tremaine, of Buffalo, says that he has given this subject some study and is quite convinced that a great deal of vivisection is unnecessary and cruel. He hopes a bill will pass restricting it. Dr. J. H. Helmer, of Lockport, who has not practised medicine certainly for the last twenty-five or thirty years, but has devoted his time chiefly to banking, says "In the name of science great cruelties have been inflicted upon animals until it has become a fad in many cases."

In another column of the JOURNAL we print in full the protest of the American Association of Obstetricians and Gynecologists at its last annual meeting, Richmond, Va., September, 1896.

While it may be proper to regulate the practice of vivisection by law so as to prevent unnecessary and unwarranted cruelty to animals, we are of the opinion that any bill drawn, as is the one under present consideration, so that it may be capable of an interpretation that will embarrass scientific research, ought not to pass. Colleges and government departments should have unrestricted privileges in this matter, and only private individuals or corporations should be controlled by law.

---

THE New York Board of Education has appointed 150 physicians to act as medical inspectors, one for each school district in the city.

THE *Ohio Medical Journal* makes the following very pertinent comments on a subject that we commend to medical educators. It is such an interesting and temperate consideration of the question that we print it entire :

ETHICS IN OUR COLLEGES.

Some one has made the suggestion that each of our medical colleges should have a chair of ethics. In one sense the suggestion is a good one. Enough would be accomplished, however, if all our colleges taught ethics both by precept and by example. There is a noteworthy difference in the behavior of men coming from different colleges. The best schools turn out an occasional scoundrel, but the percentage of honorable men turned out by some institutions is much higher than that of the graduates of others. Some colleges seem to infect their students with a propensity toward quackery, while others communicate a noble spirit of ethical conduct. Every institution should include in the curriculum a short course of lectures on ethics delivered by a member of the faculty who is above duplicity, whose life bears out the high moral precepts which it becomes his duty to inculcate upon his classes. If there is no such man in the faculty, it is time that the institution was abandoned. At the same time, it must be admitted that no amount of moral education would prevent some young men from stepping aside into "ways that are dark and tricks that are vain." For this reason, every institution should adopt measures for ascertaining the intentions of its candidates for graduation, and carefully "pluck" those who aim at no higher position than that of the charlatan. It is usually not difficult to determine this point in time to protect the institution, as well as the profession, from the disgrace that is thrown upon it by every dishonest "quacking" barnacle that infests it. A majority of each class know the intentions of each of its members. A vote of the class would, in fact, often throw light upon the worthiness of its members, for no one feels more keenly the disgrace of a degree unworthily bestowed than do the better members of the class.

